# BCN057 induces intestinal stem cell repair and mitigates radiation-induced intestinal injury

**DOI:** 10.1186/s13287-017-0763-3

**Published:** 2018-02-02

**Authors:** Payel Bhanja, Andrew Norris, Pooja Gupta-Saraf, Andrew Hoover, Subhrajit Saha

**Affiliations:** 1 Department of Radiation Oncology, The University of Kansas Medical Center, MS 4033, 3901 Rainbow Boulevard, Kansas City, Kansas 66160 USA; 2 BCN Bio Sciences, Pasadena, CA USA; 3 Department of Cancer Biology, The University of Kansas Medical Center, MS 4033, 3901 Rainbow Boulevard, Kansas City, Kansas 66160 USA

**Keywords:** Intestinal stem cell, RIGS, Abdominal radiation, Radiotherapy, Tumor

## Abstract

**Background:**

Radiation-induced gastrointestinal syndrome (RIGS) results from the acute loss of intestinal stem cells (ISC), impaired epithelial regeneration, and subsequent loss of the mucosal barrier, resulting in electrolyte imbalance, diarrhea, weight loss, sepsis, and mortality. The high radiosensitivity of the intestinal epithelium limits effective radiotherapy against abdominal malignancies and limits the survival of victims of nuclear accidents or terrorism. Currently, there is no approved therapy to mitigate radiation toxicity in the intestine. Here we demonstrate that BCN057, an anti-neoplastic small molecular agent, induces ISC proliferation and promotes intestinal epithelial repair against radiation injury.

**Methods:**

BCN057 (90 mg/kg body weight, subcutaneously) was injected into C57Bl6 male mice (JAX) at 24 h following abdominal irradiation (AIR) and was continued for 8 days post-irradiation. BCN057-mediated rescue of Lgr5-positive ISC was validated in Lgr5-EGFP-Cre-ERT2 mice exposed to AIR. The regenerative response of Lgr5-positive ISC was examined by lineage tracing assay using Lgr5-EGFP-ires-CreERT2-TdT mice with tamoxifen administration to activate Cre recombinase and thereby marking the ISC and their respective progeny. Ex vivo three-dimensional organoid cultures were developed from surgical specimens of human colon or from mice jejunum and were used to examine the radio-mitigating role of BCN057 on ISC ex vivo. Organoid growth was determined by quantifying the budding crypt/total crypt ratio. Statistical analysis was performed using Log-rank (Mantel-Cox) test and paired two-tail *t* test.

**Results:**

Treatment with BCN057 24 h after a lethal dose of AIR rescues ISC, promotes regeneration of the intestinal epithelium, and thereby mitigates RIGS. Irradiated mice without BCN057 treatment suffered from RIGS, resulting in 100% mortality within 15 days post-radiation. Intestinal organoids developed from mice jejunum or human colon demonstrated a regenerative response with BCN057 treatment and mitigated radiation toxicity. However, BCN057 did not deliver radio-protection to mouse or human colon tumor tissue.

**Conclusion:**

BCN057 is a potential mitigator against RIGS and may be useful for improving the therapeutic ratio of abdominal radiotherapy. This is the first report demonstrating that a small molecular agent mitigates radiation-induced intestinal injury by inducing ISC self-renewal and proliferation.

**Electronic supplementary material:**

The online version of this article (10.1186/s13287-017-0763-3) contains supplementary material, which is available to authorized users.

## Background

Intestinal injury is a limiting factor for definitive chemoradiation therapy of abdominal malignancies such as gastric, pancreatic, and colorectal cancer. Thus, tumoricidal doses of radiotherapy and/or chemotherapy often cannot be administered for the treatment of abdominal tumors resulting in poor survival and early metastatic spread. Moreover, radiation-induced gastrointestinal syndrome (RIGS) limits the survival of victims in a mass casualty setting from nuclear accidents or terrorism. While supportive care with antibiotics, hydration, and bone marrow transplantation can avoid death due to the hematopoietic syndrome, currently there is no approved therapy for protecting or mitigating against RIGS. Radiation doses more than 10 Gy primarily lead to gastrointestinal injury, resulting in diarrhea, dehydration, sepsis, and intestinal bleeding with eventual mortality within 10 to 15 days post-exposure [1]. A high dose of radiation induces the loss of intestinal stem cells (ISC) [2, 3] and thereby impairs epithelial regeneration. The damaged intestinal epithelium significantly reduces the mucosal integrity and promotes systemic influx of bacterial pathogens resulting in sepsis and death [2, 4]. These lethal gastrointestinal symptoms after radiation exposure are collectively known as radiation-induced gastrointestinal syndrome (RIGS), or clinically known as radiation enteritis. So far there are no Food and Drug Administration (FDA)-approved agents available to mitigate radiation-induced intestinal injury [5]. Considering the logistical barrier and unavoidable delay in treating victims in large casualty settings there is a tremendous need for therapeutic measures which can be effective even if started days after the radiation incident.

Dose-dependent radiation damage to the ISC is the primary cause of RIGS. We have reported previously that inhibition of radiation-induced ISC loss will mitigate RIGS [3]. Our recent study demonstrated that extracellular vesicle (EV)-mediated delivery of Wnt rescues ISC from radiation toxicity and induces intestinal epithelial repair with the activation of Wnt-β-catenin signaling. ISC self-renewal and proliferation, and thereby maintenance of intestinal epithelial homeostasis and repair, is primarily dependent on Wnt-β-catenin signaling [3, 6]. ISC growth factors, such as R-spondin 1 (RSPO1), activate the Wnt-β-catenin pathway to repair and regenerate the intestine following chemoradiation-induced injury [7–10]. DKK1, a negative regulator of the Wnt-β-catenin pathway, impairs the RSPO1-induced intestinal regeneration [11]. RSPO1 binds to the Lgr5 receptor [12] which is associated with the Frizzled/Lrp Wnt receptor complex [13]. Genetic deletion of *Lgr5* in mouse intestine inhibits the regenerative role of Rspo1, but epithelial regeneration can be rescued by Wnt pathway activation.

In this study we demonstrated that a small molecular agent BCN057 (3-[(Furan-2-ylmethyl)-amino]-2-(7-methoxy-2-oxo-1,2-dihydro-quinolin-3-yl)-6-methyl-imidazo[1,2-a]pyridin-1-ium) activates canonical Wnt-β-catenin signaling, mitigates RIGS, and improves survival when applied 24 h after a lethal dose of radiation exposure. BCN057 induces strong Wnt activity as demonstrated by TCF/LEF reporter assay. In an ex-vivo crypt organoid model developed from human and mice intestinal epithelium, we demonstrated that BCN057 rescued ISC from radiation toxicity and induced epithelial repair with the activation of Wnt-β-catenin signaling. However, BCN057 did not show any radioprotective effect in tumor tissue. Taken together, these observations indicate that BCN057 is an agonist of canonical Wnt-β-catenin signaling and mitigates radiation-induced intestinal injury by accelerating the repair and regeneration of ISC.

## Methods

### Pharmacokinetics

BCN057 is a novel small molecule designed with moieties targeting G protein-coupled receptors (GPCRs), and 12 mg/mL BCN057 in 30% Captisol® (β-cyclodextrin sulfobutyl ether sodium) has been formulated for subcutaneous (s.c.) administration. This formulation has shown excellent stability up to 1 year and has been well tolerated in both cell and animal use. BCN057 (mass 401.16) was administered via a single subcutaneous (s.c.) injection at the designated dose in 200 μL. Time points (post-dose) were collected by cardiac puncture in euthanized C57BL/6 mice at 0, 1, 2, 4, 6, 16, and 24 h, with three mice per time point for a total of 21 animals. Plasma samples (20 μL) were processed by a protein precipitation method. All samples were analyzed using a triple quadrupole mass spectrometer (Agilent® 6460) coupled to an HPLC system (Agilent® 1290) using a reverse-phase analytical column (Agilent® Poro Shell 300SB, C-8, 5 mm, 2.1 × 75 mm). For the analysis of BCN057, RT = 12.5 min is measured against an internal standard (3-[(Furan-2-ylmethyl)-amino]-2-(7-methoxy-2-oxo-1,2-dihydro-quinolin-3-yl)-imidazo[1,2-a]pyridin-1) with an exact mass of 387.15 and RT = 12.1 min. BCN057 is monitored with the transition from m/z 401 → 320 and quantitation is performed with the use of the internal standard yielding a linear regression least-squares fit from 2 fmol to 20 pmol with *R*^2^ = 0.99. Pharmacokinetics (PK) data were processed using PK Solutions^©^ 2.0 (Summit Research Services Montrose, CO, USA).

### Animals

Five- to 6-week-old male C57BL6/J mice, Lgr5-eGFP-IRES-CreERT2 mice, Gt(ROSA)26Sortm4(ACTB-tdTomato-EGFP)Luo/J mice, and B6.Cg-Gt(ROSA)26Sortm9(CAG-tdTomato)Hze/J mice (Jackson laboratories) were maintained ad libitum and all studies were performed under the guidelines and protocols of the Institutional Animal Care and Use Committee of the University of Kansas Medical Center. All the animal experimental protocols were approved by the Institutional Animal Care and Use Committee of the University of Kansas Medical Center (ACUP number 2016-2316).

### Development of subcutaneous tumor in mouse flank

Mice were injected subcutaneously with 1 × 10^5^ MC38 (colon carcinoma cell line) cells on the flank. About 10 days later, the tumor became palpable (3–5 mm in diameter), whereupon abdominal irradiation (AIR) of 16 Gy was delivered. Mice were divided into four groups (*n* = 10 per group): those receiving no treatment; those with AIR; those receiving BCN057; and those receiving BCN057 plus AIR. Animals received BCN057 eight times starting 24 h after AIR. Tumor measurements were performed thrice weekly using Vernier calipers along with simultaneous physical assessment of signs of systemic toxicity (malaise and diarrhea).

### Irradiation procedure

**Fig. 1:**
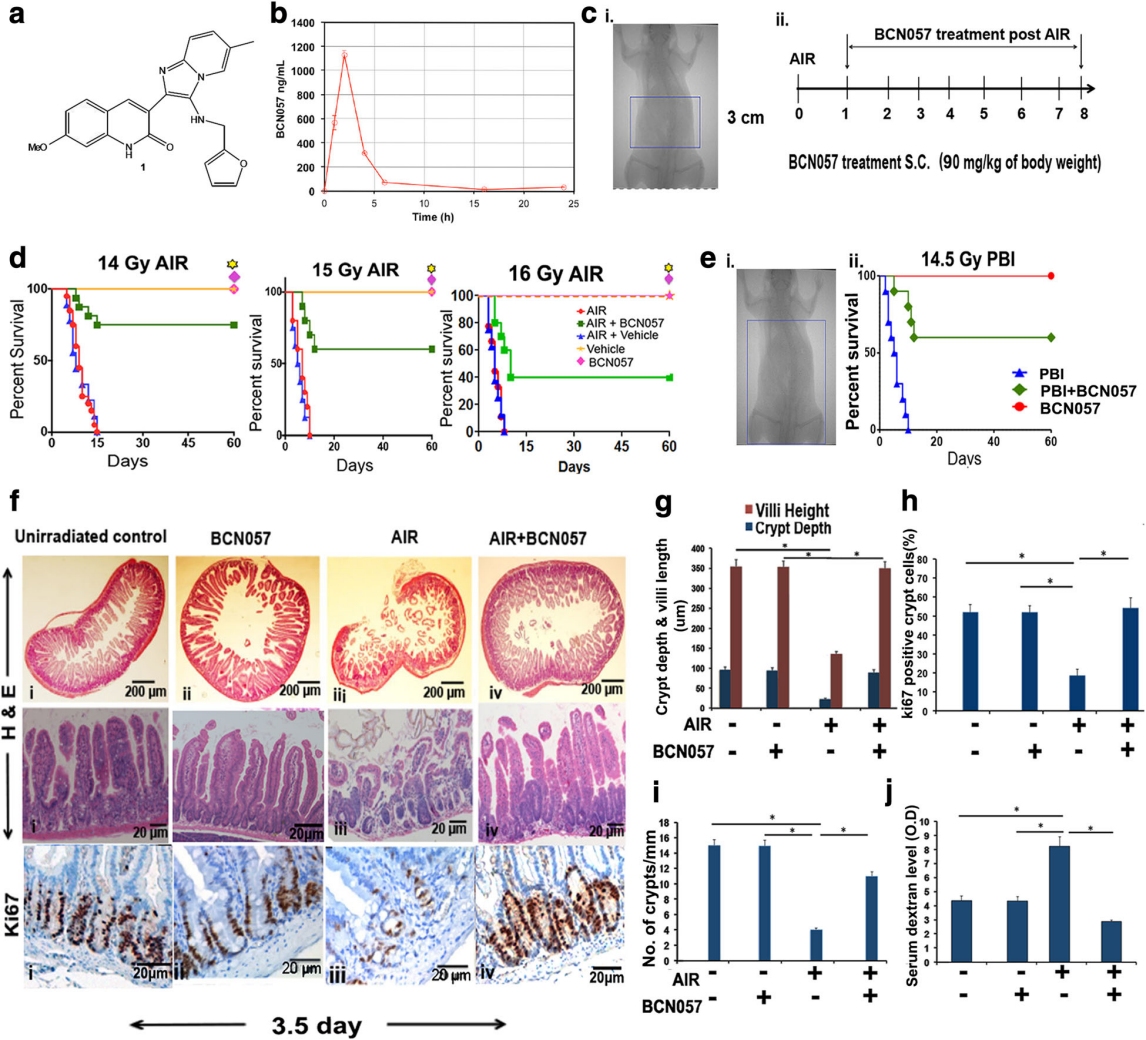
BCN057 treatment at 24 h post-irradiation mitigates RIGS and improves survival in mice. **a** Chemical structure of BCN057 (3-[(Furan-2-ylmethyl)-amino]-2-(7-methoxy-2-oxo-1,2-dihydro-quinolin-3-yl)-6-methylimidazo[1,2-a]pyridin-1-ium). **b** Pharmacokinetics of a single injection of BCN057 90 mg/kg via s.c. administration in C57BL/6 mice. C_max_ (obs) 1130.5 ng/mL, T_max_ (obs) 2.0 h, V_ss_ (expo) 15935.8 mL, CL (obs area) 700.075 mL/h. **c** Portal camera image demonstrating abdominal irradiation (AIR) exposure field (i) and BCN057 treatment schema (ii). A 3-cm area (indicated by the rectangular box) of the mouse containing the gastrointestinal tract was irradiated (irradiation field), while shielding the upper thorax, head, and neck, as well as the lower and upper extremities, and protecting a significant portion of the bone marrow, thus predominantly inducing radiation-induced gastrointestinal syndrome (RIGS). Mice exposed to AIR were treated with BCN057 (s.c.) (90 mg/kg body weight) at 24 h following irradiation and continued up to day 8 (single dose/day). **d** Kaplan-Meier survival analysis of C57BL/6 mice (*n* = 25/group) receiving vehicle, BCN057, or no treatment at 24 h after AIR (14 Gy, 15 Gy, or 16 Gy) and continued up to day 8. Mice receiving BCN057 after 14 Gy, 15 Gy, or 16 Gy AIR demonstrated 80%, 60%, and 40% survival, respectively, and they continued to survive beyond 60 days without any symptoms of gastroenteritis or any other health complications, whereas mice receiving vehicle or no treatment following AIR died within 15 days (*p* < 0.0003, *p* < 0.0004, and *p* < 0.0007, respectively, log-rank (Mantel-Cox) test). BCN057 or vehicle do not confer any toxicity to normal mice. **e** (i) Kaplan-Meier survival analysis of C57BL/6 mice (*n* = 25/group) exposed to partial body irradiation (PBI). Head, neck, and upper extremities were shielded to spare 40% of bone marrow (BM40%). The part of the body exposed to irradiation is indicated by a rectangular box. (ii) Mice receiving BCN057 at 24 h post-PBI demonstrated 70% survival compared with untreated controls (*p* < 0.0001 log-rank (Mantel-Cox) test). **f** H&E stained representative cross section of jejunum from C57BL/6 mice treated with BCN057 at 24 h post-AIR (upper panels). Note restitution of the epithelium in mice receiving BCN057 compared with irradiated controls. H&E stained representative transverse section of jejunum from C57BL/6 mice treated with BCN057 at 24 h post-AIR (middle panels). Note restitution of crypt villus structure in BCN057-treated mice. However, irradiated, untreated mice showed significant loss of crypts along with villi denudation. Representative Ki67 immunohistochemistry of mice jejunal sections (lower panels). Note the increase in Ki67-positive crypt cells in mice receiving BCN057 at 24 h after AIR (iv) compared with AIR controls (ii). **g–i** Histogram showing crypt depth and villi length (**g**), percentage of Ki67-positive crypt cells (**h**), and number of crypts per mm (**i**) in the jejunum. Irradiated mice receiving BCN057 at 24 h after AIR demonstrated an increase in crypt depth and villi length (*p < 0.0006), number of crypts (*p < 0.0004), and percentage of Ki67-positive crypt cells (*p < 0.0005) compared with irradiated controls. **j** Histogram demonstrating serum dextran level in C57BL/6 mice exposed to AIR and then treated with or without BCN057. Mice receiving BCN057 treatment demonstrated lower serum dextran levels, thereby suggesting restitution of epithelial integrity compared with irradiated untreated controls (*p < 0.004, *n* = 3 per group, unpaired *t* test, two-tailed). Unirradiated control mice and unirradiated BCN057 treated mice also showed lower serum dextran level compared with irradiated controls (*p < 0.006, *p < 0.005, unpaired *t* test, two-tailed)

AIR was performed on anesthetized mice (with a continuous flow of 1.5 mL/min 1.5% isoflurane in pure oxygen) using the small animal radiation research platform (SARRP; XStrahl, Surrey, UK). A 3-cm area of the mice containing the gastrointestinal tract (GI) was irradiated (Fig. 1c), thus shielding the upper thorax, head, and neck, as well as the lower and upper extremities, and protecting a significant portion of the bone marrow, thus predominantly inducing RIGS. A radiation dose of 14–15 Gy was delivered to the midline of the GI, ensuring homogeneous delivery by performing half of the total irradiation from the anterior-posterior direction and the second half from the posterior-anterior direction. Partial body irradiation (PBI) was delivered to mice after shielding the head and fore limbs where 40% of the total bone marrow was exposed (BM40) to irradiation (Fig. 1e) [14]. The total irradiation time to deliver the intended dose was calculated with respect to dose rate, radiation field size, and fractional depth dose to ensure accurate radiation dosimetry.

### TCF/LEF (TOPFLASH) reporter assay

To determine the canonical Wnt activity of BCN057, HEK293 cells (Signosis, Santa Clara, CA, USA) with a TCF/LEF luciferase reporter construct were treated with BCN057 or vehicle control or phosphate-buffered saline (PBS). Lithium chloride (LiCl; 10 mM) treatment was used as a positive control for luciferase activity. Luciferase activity was determined after 24 h using the Dual-Luciferase Reporter Assay System (Promega) as per the manufacturer’s protocol. HEK293 cells with a FOPFLASH construct (mutated TCF/LEF-binding site), were used as a negative control. The HEK293 (human embryonic kidney) cell line was routinely characterized in the laboratory based on morphology and gene-expression patterns. Cells were confirmed to be free of mycoplasma contamination.

### Histology

Since radiation doses > 8 Gy induce cell cycle arrest and apoptosis of the crypt epithelial cells within day 1 post-radiation, resulting in a decrease in regenerating crypt colonies by day 3.5 and ultimately villi denudation by day 7 post-radiation exposure, animals were euthanized when moribund or at 3.5 days after AIR for time-course experiments, and intestines were collected for histology (Additional file 1: Supplement method).

### Crypt proliferation rate

To visualize the villous cell proliferation, mid-jejunum was collected for paraffin embedding and Ki67 immunohistochemistry. Tissue sections were routinely deparaffinized and rehydrated through graded alcohols and incubated overnight at room temperature with a monoclonal anti-Ki67 antibody (M7240 mib1; Dako). Nuclear staining was visualized using streptavidin-peroxidase and diaminobenzidine (DAB) and samples were lightly counterstained with hematoxylin. Murine crypts were identified histologically according to the criteria reported previously [15] (Additional file 1: Supplement method). To detect the presence of Ki67 in Lgr5-positive ISC, jejunal sections from Lgr5-eGFP-IRES-CreERT2 mice were stained with rabbit polyclonal antibody to Ki67 (Abcam, #ab15580; dilution 1:250) followed by secondary antibody donkey anti-Rabbit Alexa fluor 647 (Life technologies, #A31573; dilution 1:1000). Nuclei was counterstained with DAPI.

### Determination of villi length and crypt depth

The crypt depth was independently and objectively analyzed and quantitated in a blinded manner from coded digital photographs of crypts from hematoxylin and eosin (H&E) stained slides using ImageJ 1.37 software to measure the height in pixels from the bottom of the crypt to the crypt villus junction. Villi length was determined by measuring the length from the crypt villus junction to the villous tip. This measurement (in pixels) was converted to length (in μm) by dividing with the following a conversion factor (1.46 pixels/μm).

### Detection of apoptosis in situ

Apoptotic cells were detected in situ by performing TdT-mediated digoxigenin-labeled dUTP nick-end labeling (TUNEL) staining. Briefly, paraffin embedded sections were de-paraffinized, rehydrated through graded alcohols, and stained using an ApopTag kit (Intregen Co., Norcross, Georgia, USA). The apoptotic rate in crypt cells was quantified by counting the percentage of apoptotic cells in each crypt with analysis restricted to “intact” longitudinal crypt sections in which the base of the crypt was aligned with all the other crypt bases and the lumen.

### β-catenin immunohistochemistry of mouse jejunum

β-Catenin immunohistochemistry was performed in paraffin-embedded sections of mouse jejunum [16]. In brief, tissue was stained using the anti-β-catenin antibody (1:100 dilution; BD Transduction Laboratories, Franklin Lakes, NJ; #610154) at room temperature for 2 h followed by staining with horseradish peroxidase-conjugated anti-mouse antibody (Dako, Denmark) at room temperature for 1 h. Nuclei were counter-stained with hematoxylin (blue). β-Catenin-positive nuclei (stained dark brown) were calculated from 15 crypts per field, and five fields per mice (Additional file 1: Supplement method).

### Real-time polymerase chain reaction to determine the expression of β-catenin target genes and intestinal stem cell markers in crypt epithelium

To compare the mRNA levels of β-catenin target genes in intestinal crypt cells from irradiated mice treated with BCN057 or PBS, real-time polymerase chain reaction (PCR) was performed for the genes *Ephb2*, *Ascl2*, *Olf*, *Tcf-4*, *Lef1*, *Sox9*, and *Axin2* using the Wnt target gene quantitative PCR (qPCR) primers (Additional file 2: Table S1). The expression of the intestinal stem cell markers LGR5, K19, HES-1, and CD44 were determined by real-time PCR using the primer pairs listed in Additional file 3 (Table S2). Total RNA was extracted using TRIzol kit (Invitrogen, CA, USA). A detailed protocol is described in the supplementary methods section (Additional file 1: Supplement method).

### FITC-dextran permeability assay

At day 5 post-AIR, animals were gavaged with 0.6 mg/g body weight of an FITC-dextran solution (4000 kD size; Sigma). Four hours after gavage, mice were killed and serum was obtained by cardiac puncture [17]. Samples were measured in a 96-well plate using a Flexstation II 384 multiwell fluorometer (Molecular Devices). A standard curve was constructed using mouse serum having increasing amounts of FITC-dextran to determine the serum levels of FITC-dextran in different treatment groups.

### In vitro culture of intestinal crypt organoids

Small intestine from Lgr5-eGFP-IRES-CreERT2 and R26-ACTB-tdT-EGFP mice, or their littermate control mice, and malignant/non-malignant colon tissue from human surgical specimens was used for Crypt isolation and development of ex vivo organoid culture [18–20]. Lgr5-eGFP-IRES-CreERT2 mice were crossed with Gt(ROSA)26Sortm4(ACTB-tdTomato-EGFP)Luo/J mice (Jackson Laboratories) [21]. In Gt(ROSA)26Sortm4(ACTB-tdTomato-EGFP)Luo/J mice tdTomato is constitutively expressed (independent of Cre recombination) in the membrane of all cells, and therefore allows better visualization of cellular morphology. Human tissues were received from the University of Kansas Medical Center Biorepository (HSC #5929). A detail protocol is described in the supplementary methods section (Additional file 1: Supplement method).

### In vivo lineage tracing assay

Lgr5-eGFP-IRES-CreERT2 mice were crossed with B6.Cg-Gt(ROSA)26Sortm9(CAG-tdTomato)Hze/J mice (Jackson Laboratories) [22] to generate the Lgr5-eGFP-IRES-CreERT2; Rosa26-CAG-tdTomato heterozygote. To examine the contribution of Lgr5 ISC to tissue regeneration under steady-state conditions, lineage tracing was induced by tamoxifen administration in Cre reporter mice to mark the ISC and their respective tdT-positive progeny. Adult mice were injected with tamoxifen (Sigma; 9 mg per 40 g body weight, intraperitoneally) to label Lgr5^+^ lineages. For irradiation injury studies, mice were given 14–15 Gy AIR, and tissue was harvested on day 8 post-irradiation.

### NCI 60 Cancer Cell Line Screen

The NCI 60 Cancer Cell Line Screen was performed according to the protocol described previously [23–25]. Briefly, 100 μL of each cell preparation was tested in accordance with its particular type and density, ranging from 5000–40,000 cells per well in a 96-well microtiter plate, corresponding to their own growth rate. BCN057 was evaluated at 10 μM with incubation for 48 h in a 5% CO_2_ atmosphere with 100% humidity. Proliferation was assayed using the sulforhodamine B assay [26, 27] with a plate reader to read the optical densities.

### Statistics

Mice survival/mortality in the different treatment group was analyzed by Kaplan-Meier statistics as a function of radiation dose using Graphpad Prism 6.0 software for Mac. Mice were sorted randomly after genotyping to each experimental and control group. The minimum number of mice used for survival/mortality study was *n* = 25 per group. For histopathological analysis, jejunal sampling regions were chosen at random for digital acquisition for quantitation. Digital image data were evaluated in a blinded manner as to treatment. A two-sided student’s *t* test was used to determine significant differences between experimental cohorts (*P* < 0.05) with representative standard errors of the mean (SEM).

## Results

### BCN057 mitigates RIGS and improves survival following a lethal dose of radiation

Lethality from acute radiation syndrome (ARS) depends upon dose-dependent injury to various organs. Total body exposure to a radiation dose higher than 10 Gy results in mortality within 15 days post-exposure primarily due to RIGS. Intestinal epithelium is highly radiosensitive because of its rapid self-renewal rate compared with any other organ. Every 4–5 days a new epithelium takes charge of mucosal defense under very strict epithelial homeostasis. A high dose of radiation disrupts this homeostatic balance, kills ISC, and impairs the repair process resulting in complete loss of the mucosal barrier within 5–10 days post-exposure.

In this study, we have demonstrated that BCN057 mitigates RIGS and improves survival of mice exposed to a lethal dose of irradiation. Initially, pharmacokinetic studies of BCN057 were performed to examine time-dependent plasma exposure parameters for the subcutaneous route of administration (Fig. 1b). BCN 057 showed plasma exposure over 24 h with a rapid C_max_ at approximately 2 h and complete clearance over 24 h. Consequently, one dose was given every 24 h for 8 days (Fig. 1c) as a dose regimen. To examine the radio-mitigating role of BCN057 against RIGS, C57BL/6 mice were exposed to graded doses of AIR (14–16 Gy) after shielding the thorax, head, neck, and extremities, thus protecting the bone marrow (Fig. 1c) [4, 7]. A single fraction of 14, 15 or 16 Gy AIR induces RIGS and lethality in 100% of animals within 7–14 days post-exposure. Mice receiving BCN057 at 24 h post-AIR continued to survive beyond 30 days post-exposure without showing any symptoms of RIGS (Fig. 1d). These results clearly indicate that BCN057 mitigates the lethal radiation injury in the intestine.

In the event of accidental radiation, it is highly probable that many other organ systems will also be exposed and their differential responses to various doses of irradiation will impact the gastrointestinal acute radiation syndrome (GI-ARS) dose response. Involvement of bone marrow will have a major impact on GI-ARS, primarily regarding intestinal inflammation and mucosal immunity to mitigate infection resulting from bacterial translocation through an impaired intestinal mucosal barrier. To understand the involvement of bone marrow in survival outcome on BCN057 treatment, C57BL/6 mice were exposed to partial body irradiation (PBI) where 40% of the total bone marrow was exposed (BM40) to irradiation after shielding the head and forelimbs (Fig. 1e) [14]. Treatment with BCN057 at 24 h post-exposure 14.5 Gy PBI rescued 60% of mice from radiation lethality (*p* < 0.0001). However, all the untreated mice were dead within 12 days post-exposure (Fig. 1e). These data indicate that BCN057 can rescue GI epithelium from radiation lethality even in the absence of a protective function of bone marrow.

We continued to observe these BCN057-treated mice following AIR/PBI up to day 60 post-exposure. These mice did not develop any clinical conditions, indicating complete cure with BCN057 treatment. Histopathological analysis of mice jejunum at 3.5 days post-AIR clearly demonstrated a loss of crypts with significant denudation of villus length, indicating that RIGS is the primary cause of death. Mice receiving BCN057 treatment demonstrated normal crypt villus structure with an increase in the number of crypts and preserved villous length (Fig. 1f, g, i). The percentage of Ki67 crypt epithelial cells was significantly higher in BCN057-treated mice compared with untreated irradiated controls (Fig. 1f, h; *p* < 0.0005). However, treatment with BCN057 in non-irradiated mice did not induce any changes in crypt villus morphology or Ki67-positive cells (Fig. 1f, h) compared with unirradiated controls.

Since dextran is unable to cross the GI epithelia unless it is compromised, dextran in the blood is a good indicator of epithelial damage [28]. Blood FITC-dextran levels were measured at 4 h after gavage. Treatment with BCN057 significantly reduced the FITC-dextran uptake in the blood stream in irradiated mice compared with untreated irradiated control mice (*p* < 0.004, unpaired *t* test, two-tailed; Fig. 1j). These data indicate restitution of intestinal epithelial integrity by BCN057 treatment.

### BCN057 activates β-catenin in irradiated jejunum

**Fig. 2:**
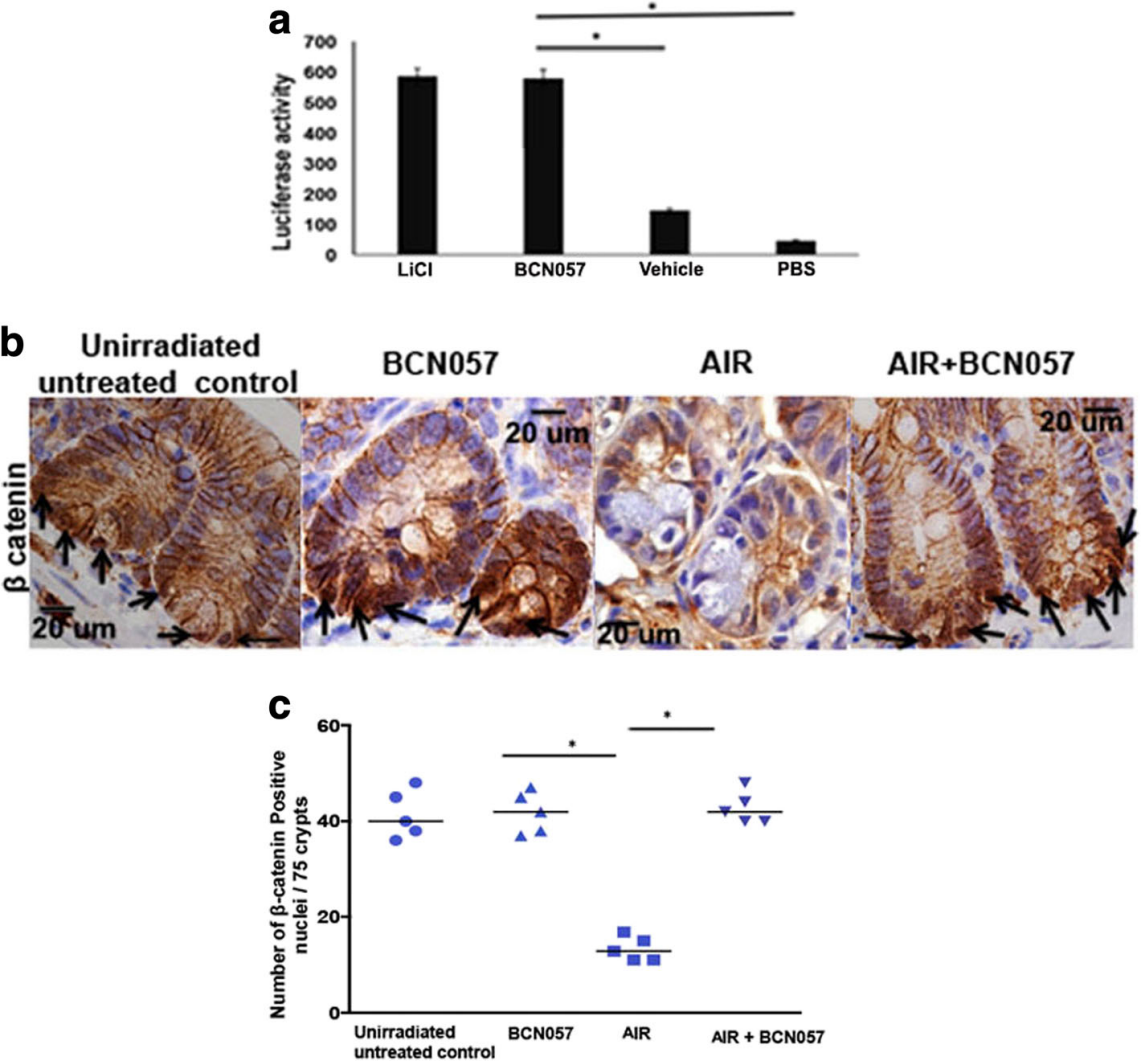
BCN057 activates Wnt-β-catenin signaling. **a** HEK293 cells having TCF/LEF luciferase reporter construct were treated with BCN057 or LiCl. Treatment with BCN057 showed higher luciferase activity compared with vehicle control (*p < 0.001). **b** Representative microscopic images (×60 magnification) of jejunal sections immunostained with anti β-catenin antibody to determine β-catenin nuclear localization. Nuclei stained with hematoxylin. Irradiated mice treated with BCN057 demonstrated more nuclear β-catenin staining (dark brown; indicated with arrows) at the base of the crypt compared with control nuclei stained blue. **c** Nuclear β-catenin count. Each data point is the average of the number of β-catenin-positive nucleus from 15 crypts per field, five fields per mice. The number of β-catenin-positive nuclei in irradiated mice receiving BCN057 is higher compared with irradiated controls (*p < 0.005). Unirradiated mice receiving BCN057 showed a higher number of β-catenin-positive nuclei compared with irradiated controls (*p < 0.001; unpaired *t* test, two-tailed). AIR abdominal irradiation, PBS phosphate-buffered saline

Intestinal epithelial self-renewal, homeostasis, and repair are dependent upon Wnt-β-catenin signaling. Activation of Wnt-β-catenin signaling translocates β-catenin to the nucleus to switch on a series of gene expressions that support ISC maintenance and proliferation [7]. The Wnt activity of BCN057 was first examined by TCF/LEF reporter assay. Graded doses of BCN057 demonstrated a significant increase in the luciferase signal compared with vehicle controls, indicating Wnt activity of BCN057 (*p* < 0.001; Fig. 2a).

We then analyzed the effect of BCN057 on crypt epithelial β-catenin activation. Immunohistochemical analysis of jejunal sections from non-irradiated mice showed characteristic β-catenin with 40 ± 5 cells being positive for nuclear β-catenin per 75 crypts (Fig. 2b, c). Mice exposed to AIR (16 ± 2) had significantly fewer nuclear β-catenin-positive cells compared with unirradiated controls at day 3.5 post-AIR. However, mice receiving BCN057 at 24 h post-AIR demonstrated a significant increase in nuclear β-catenin-positive cells compared with irradiated untreated animals (Fig. 2b, c). Nuclear β-catenin-positive cells were primarily observed in the crypt bottom which is also the location for ISC, indicating activation of Wnt-β-catenin signaling in ISC. PCR array analysis of β-catenin target genes in crypt epithelial cells also showed several fold increases in the mRNA levels in irradiated mice treated with BCN057 compared with irradiated controls (Table 1). In summary, these data suggest that BCN057 activates the Wnt-β-catenin signaling in the irradiated crypt to induce crypt stem cell proliferation and regeneration.

**Table 1:** Wnt target gene expression

Gene name	Fold change
*Ephb2*	1.68 ± 0.03
*Ascl2*	4.9 ± 0.1
*Olf2*	6.4 ± 0.2
*Tcf4*	2.4 ± 0.02
*Lef1*	4.7 ± 0.05
*Sox9*	3.22 ± 0.2
*Axin2*	5.3 ± 0.6

### BCN057 rescues Lgr5^+^ ISC from radiation toxicity

**Fig. 3:**
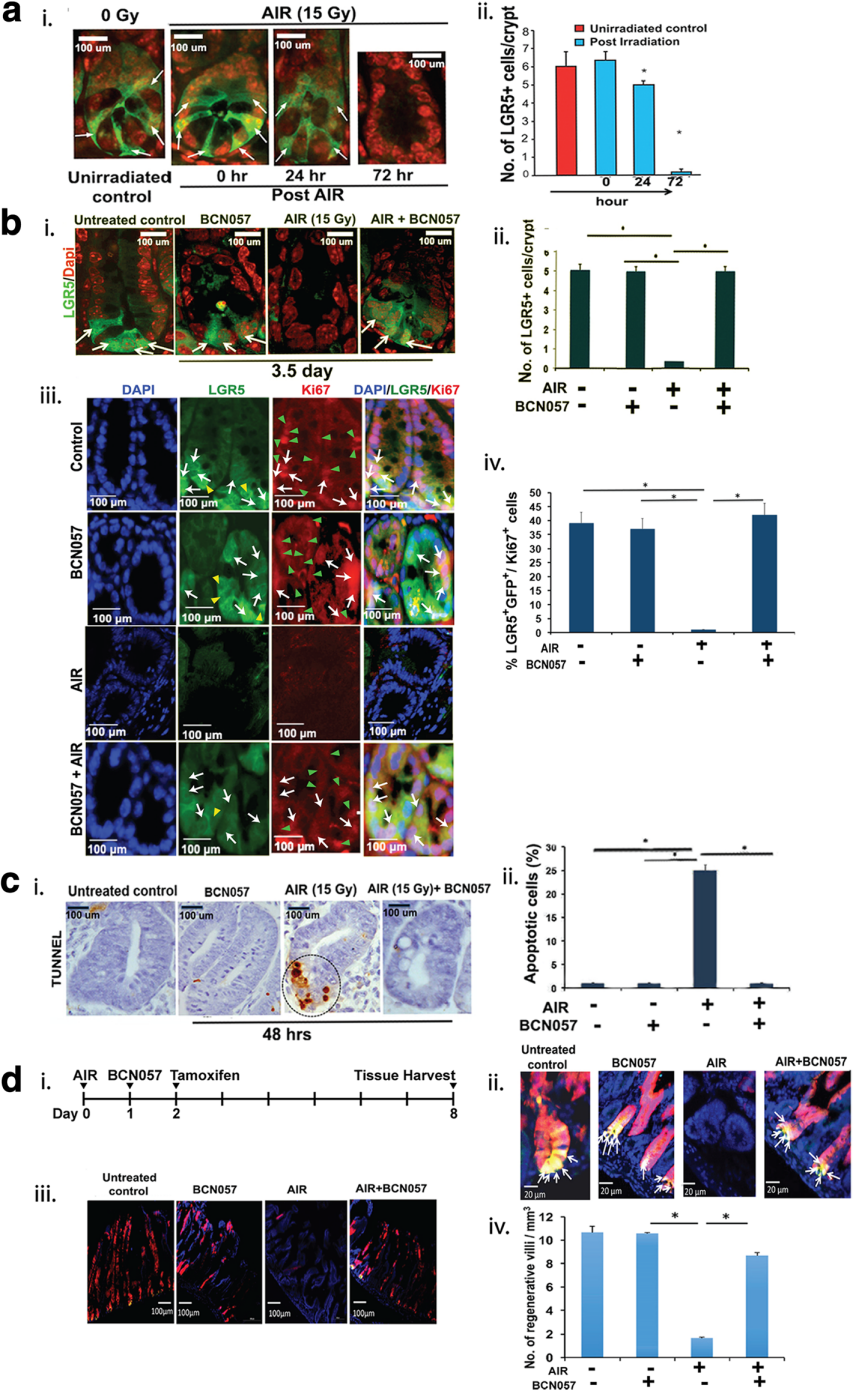
BCN057 rescued Lgr5^+^ ISC and induced a regenerative response in vivo. **a** (i) Time-course study on the effect of abdominal irradiation (AIR) on Lgr5-positive ISC. Representative images of jejunal sections demonstrating the presence of green fluorescent protein (GFP)^+^Lgr5^+^ ISC (indicated with arrow) in Lgr5/GFP-IRES-Cre-ERT2 knock-in mice up to 24 h post-AIR. All the ISC at the crypt base disappeared at 72 h post-AIR. (ii) The number of Lgr5^+^GFP^+^ ISC per crypt in jejunal sections from Lgr5/GFP-IRES-Cre-ERT2 knock-in mice at different time points post-AIR. The number of Lgr5^+^GFP^+^ ISC per crypt reduced at 24 h post-irradiation (*p < 0.04). At 72 h post-AIR, most of the Lgr5^+^GFP^+^ ISC disappeared (*p* < 0.0001). **b** (i) Representative images of jejunal sections at 3.5 days post-AIR demonstrating the presence of GFP^+^Lgr5^+^ ISC (indicated with arrow) in Lgr5/GFP-IRES-Cre-ERT2 knock-in mice receiving BCN057 at 24 h post-AIR. Note the absence of GFP^+^ cells in mice receiving only AIR. (ii) The number of Lgr5^+^GFP^+^ ISC per crypt in jejunal sections from Lgr5/GFP-IRES-Cre-ERT2 knock-in mice exposed to irradiation and then treated with BCN057. The number of Lgr5^+^ cells are significantly higher in BCN057-treated irradiated mice compared with AIR controls (*p < 0.0001). Unirradiated mice receiving BCN057 also demonstrated a higher number of Lgr5^+^ cells at the crypt base compared with AIR controls (*p < 0.0002; unpaired *t* test, two-tailed). (iii) Representative images of jejunal sections demonstrating the presence of Ki67 in Lgr5^+^GFP^+^ ISC localized in the crypt base. Representative images from the single fluorescence channel showed localization of Lgr5^+^GFP^+^ cells (green, indicated with yellow arrow head) and Ki67^+^ cells (red, indicated with green arrow head). Cells that are double-positive for Ki67 and GFP are indicated with white arrows in both the single fluorescence channel and in the merged image. (iv) The percentage of Lgr5^+^GFP^+^/Ki67^+^ in jejunal sections from Lgr5/GFP-IRES-Cre-ERT2 knock-in mice exposed to irradiation and then treated with BCN057. The percentage of Lgr5^+^GFP^+^/Ki67^+^ cells are significantly higher in BCN057-treated irradiated mice compared with AIR controls (*p < 0.0001). Unirradiated mice receiving BCN057 also demonstrated a higher percentage of Lgr5^+^GFP^+^/Ki67^+^ cells at the crypt base compared with AIR controls (*p < 0.0003; unpaired *t* test, two-tailed). **c** (i) Representative image of jejunal sections at 48 h post-AIR demonstrating the presence of TUNEL-positive apoptotic cells at the crypt base in mice exposed to AIR. However, mice receiving the BCN057 treatment at 24 h post-AIR did not show any TUNEL-positive cells. (ii) Percentage of TUNEL-positive apoptotic cells in jejunal sections from mice exposed to AIR. The percentage of TUNEL-positive cells are significantly higher in the AIR group compared with mice receiving BCN057 at 24 h post-AIR (*p* < 0.0002). **d** (i) Schematic representation of the treatment schema for lineage tracing assay in Lgr5-eGFP-IRES-CreERT2; Rosa26-CAG-tdTomato mice. (ii) Confocal microscopic images (×40) of the jejunal section from Lgr5-eGFP-IRES-CreERT2; Rosa26-CAG-tdTomato mice. tdTomato (tdT)-positive cells are shown in red; Lgr5^+^GFP^+^ cells are shown in green. Nuclei are stained with DAPI (blue). Marked expansion of tdT-positive red cells above the +4 position (representing transit amplifying cells) were noted with BCN057 treatment. Please note the presence of yellow cells at the bottom of the crypt representing tdT-positive and GFP^+^Lgr5^+^ ISC (yellow due to red + green). (iii) Confocal microscopic images (×10) of the jejunal section from Lgr5-eGFP-IRES-CreERT2; Rosa26-CAG-tdTomato mice. Please note the presence of villi containing red tdT-positive cells (regenerative villi) in unirradiated controls or BCN057-treated mice. In the absence of BCN057 treatment, no tdT-positive cells were noted in irradiated mice jejunum. (iv) The number of regenerative villi. Irradiated mice receiving BCN057 showed a significantly higher number of regenerative villi compared with irradiated controls (*p < 0.002). Un-irradiated mice receiving BCN057 also demonstrated a higher number of regenerative villi compared with irradiated controls (*p < 0.0006)

To study this effect in vivo we examined the role of BCN057 on ISC survival by exposing Lgr5/GFP-IRES-Cre-ERT2 knock-in mice to 15 Gy AIR and then treatment with BCN057. A time-course study demonstrated that Lgr5^+^GFP^+^ ISC were present up to 24 h post-irradiation but disappeared thereafter from the crypt base (Fig. 3a). TUNEL staining demonstrated that most of the crypt base columnar cells were apoptotic at 48 h post-AIR (Fig. 3c). Irradiated mice receiving BCN057 at 24 h post-irradiation showed significant preservation of Lgr5-positive ISC (*p* < 0.001; Fig. 3b) with a significant reduction in radiation-induced apoptosis in crypt base columnar cells (Fig. 3c). However, mice receiving a first dose of BCN057 at 72 h post-irradiation could not induce repair of the intestinal epithelium (Additional file 4: Figure S1), possibly due to the absence of ISC. This result suggests a potential window of opportunity up to 24 h post-irradiation to mitigate radiation-induced damage in the intestine.

Treatment with BCN057 at 24 h post-irradiation activates proliferation of Lgr5-positive ISC in irradiated intestinal epithelium. Ki67 staining on jejunal sections from Lgr5-EGFP-ires-CreERT2 mice demonstrated that Lgr5-positive ISC are also positive for Ki67 in response to BCN057 treatment (Fig. 3b). Lineage tracing assay using Lgr5-EGFP-ires-CreERT2-R26-CAG-tdT mice [29] demonstrated that BCN057 induces the regenerative response of Lgr5-positive ISC. In this mouse model, tamoxifen-mediated activation of cre-recombinase (Fig. 3d) under the Lgr5 promoter expresses tdTomato in epithelial cells derived from Lgr5-positive ISC. Therefore, quantification of these tdTomato (tdT)-positive cells in irradiated epithelium with or without BCN057 determines the regenerative response of Lgr5-positive ISC. Tamoxifen treatment in the AIR + BCN057 group demonstrated the presence of tdT-positive cells in the crypt epithelium (Fig. 3d; Additional file 5: Figure S2). However, in irradiated untreated mice, tdT-positive cells are absent, suggesting the loss of regenerative capacity of Lgr5-positive ISC (Fig. 3d). We have quantified the number of villi containing tdT-positive red cells (regenerative villi) for a comparison between the AIR and AIR + BCN057 treatment groups. BCN treatment after AIR results in a significant increase in regenerative villi compared with untreated irradiated controls (*p < 0.002; Fig. 3d). All this evidence clearly demonstrates that BCN057 induces the repair process of the intestinal epithelium by inducing the growth and proliferation of intestinal stem cells.

**Fig. 4:**
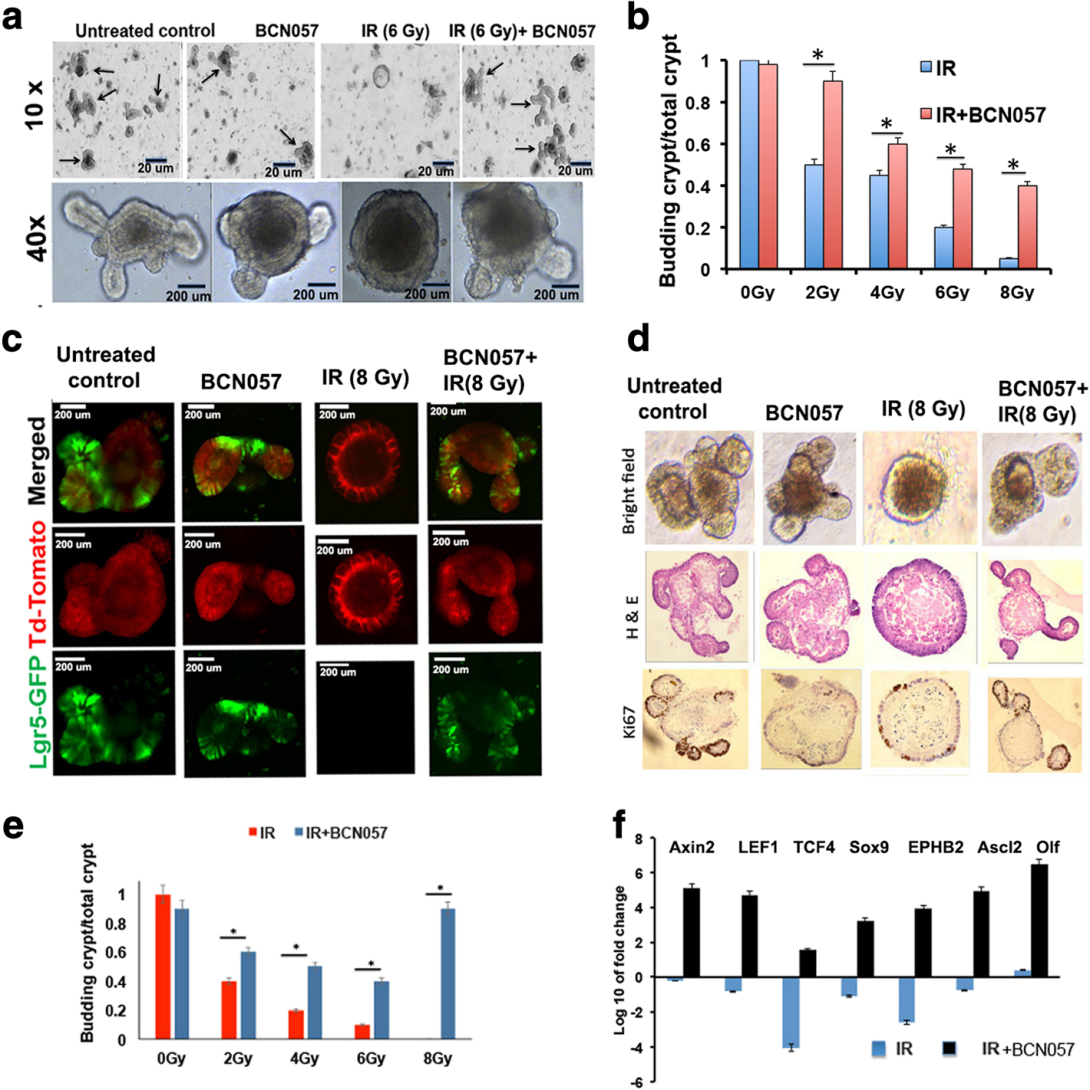
BCN057 mitigates radiation toxicity in intestinal organoids derived from mice and human tissue. **a** Microscopic images (phase contrast) of intestinal organoids along with **b** a histogram of budding crypt/total crypt ratio demonstrating that BCN057 treatment improves organoid growth compared with irradiated (IR) controls (2 Gy, *p < 0.006; 4 Gy, *p < 0.008; 6 Gy, *p < 0.003; 8 Gy, *p < 0.002). Images at 10× (indicated with arrow) and 40× demonstrated the presence of a budding crypt in irradiated organoids treated with BCN057. **c** Confocal microscopic images of organoids developed from Lgr5-EGFP-CRE-ERT2; R26- ACTB-tdTomato-EGFP mice demonstrated that BCN057 treatment increases the presence of Lgr5-positive cells (green) in budding crypts compared with irradiated controls. tdTomato is constitutively expressed in these mice as membrane-bound protein, and therefore allows better visualization of cellular morphology. **d** Microscopic image (phase contrast) of organoids developed from human non-malignant colon demonstrating the loss of crypt domain exposed to irradiation (8 Gy). Both bright field and hematoxylin and eosin (H&E) staining demonstrated complete loss of budding crypt at 72 h post-irradiation. However, BCN057 treatment at 1 h post-radiation rescued the organoids from radiation toxicity and accelerated crypt-villus budding. Note the presence of a budding crypt-like structure in the BCN057-treated group. Ki67 staining demonstrated positive staining in the budding crypt-like structure in the BCN057-treated organoids indicating cell proliferation in this group. However, irradiated untreated organoids did not show any Ki67-positive budding crypt-like structure. **e** Ratio of budding crypts to total number of crypts in human colonic organoids is increased with BCN057 treatment in irradiated organoids compared with untreated irradiated controls (2 Gy, *p < 0.009; 4 Gy, *p < 0.006; 6 Gy, *p < 0.001; 8 Gy, *p < 0.003; unpaired *t* test, two-tailed). **f** qPCR analysis demonstrated that BCN057 treatment increases the mRNA levels of Wnt target genes in epithelial cells isolated from irradiated human colonic organoids. Data are the average of three human subjects

To further analyze the specific effect of BCN057 on the ISC population, we developed an ex vivo intestinal organoid culture system [3] exposed to graded doses of irradiation. Treatment with BCN057 (10 μM) at 1 h after irradiation rescued the organoids from radiation toxicity and improved the ratio of budding crypt/total crypt (Fig. 4a, b). Intestinal crypts were isolated from Lgr5/EGFP-IRES-Cre-ERT2; R26-ACTB-tdTomato-EGFP mice to allow the visualization of the ISC. At a dose level of 8 Gy most of the Lgr5-positive ISC had disappeared within 48 h resulting in a significant loss in budding crypts with changes in existing crypt morphology indicating inhibition of ISC growth and proliferation in response to radiation exposure (Fig. 4c). Treatment with BCN057 (10 μM) at 1 h after irradiation rescued the organoids from radiation toxicity and improved the growth proliferation of Lgr5-positive ISC (Fig. 4c).

### BCN057 mitigates radiation injury in human colonic epithelium-derived organoids

To examine the effect of BCN057 on human intestinal epithelial tissue, surgical specimens collected from normal colon at least 10 cm apart from the malignant site were used to develop an ex vivo crypt organoid. At a dose level of 8 Gy all the budding crypts have disappeared in the organoids. However, organoids treated with BCN057 at 1 h post-irradiation had budding crypts with complete restitution of organoid structure (Fig. 4d). Quantification of the budding crypt-like structure demonstrated a higher number of budding crypts/total crypt ratio with BCN057 treatment compared with irradiated controls, suggesting improvement in growth and proliferation in organoids receiving BCN057 (Fig. 4e). BCN057-treated organoids demonstrated an increase in mRNA levels of intestinal stem cell-specific markers such as LGR5, K19, CD44, and HES1 (*p* < 0.001, *p* < 0.005, *p* < 0.001, and *p* < 0.004, respectively) compared with irradiated untreated organoids (Table 2). We have also evaluated the effect of BCN057 on mRNA levels of β-catenin target genes in human colonic organoids. Organoids exposed to irradiation and then treated with BCN057 demonstrated a several-fold increase in expression of β-catenin target genes, indicating activation of Wnt-β-catenin signaling (Fig. 4f).

**Table 2:** Intestinal stem cell-specific marker gene expression

Gene name	Fold change
*Lgr5*	4.2 ± 0.02
*K19*	2.3 ± 0.05
*CD44*	3.8 ± 0.1
*Hes1*	4.7 ± 0.05

### BCN057 does not protect malignant tissue from radiation

**Fig. 5:**
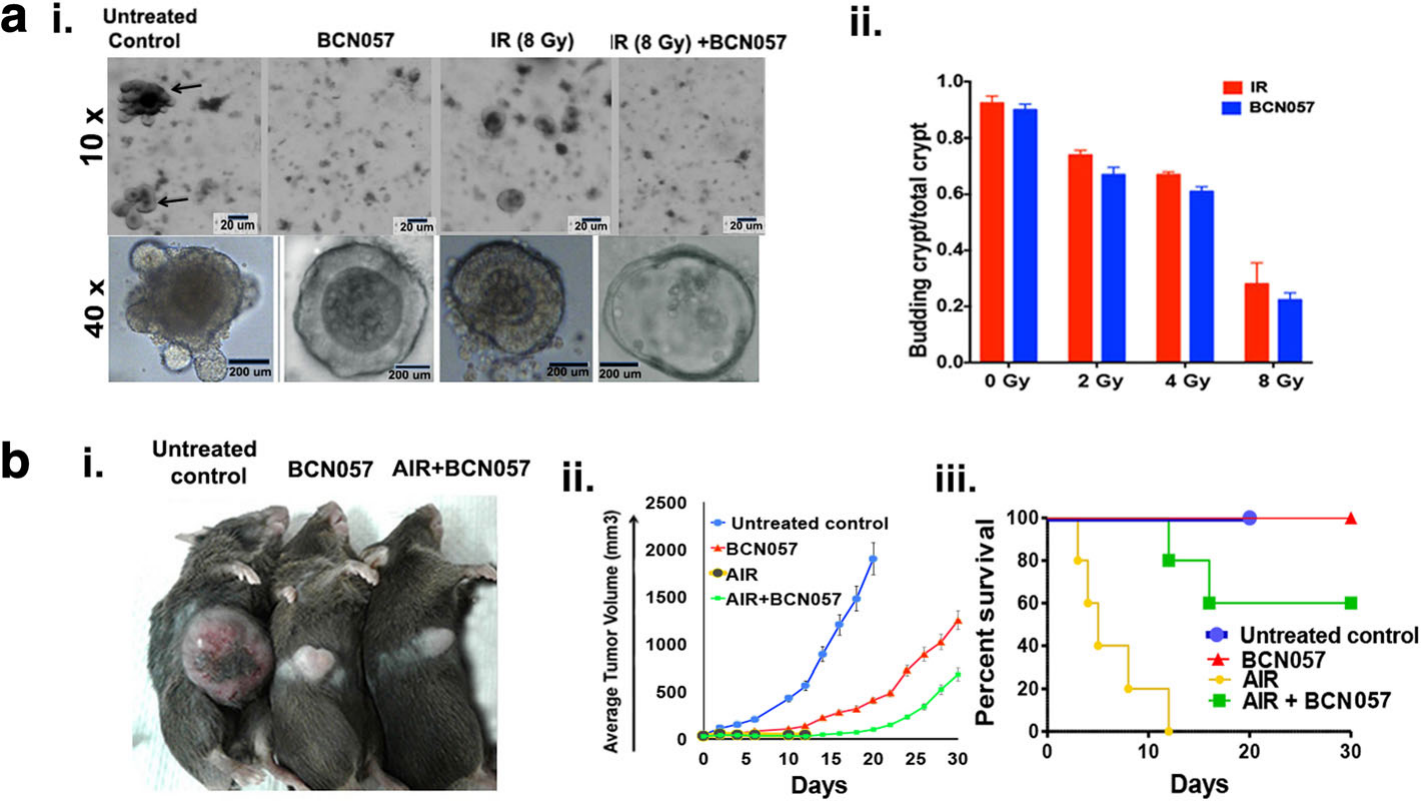
BCN057 does not have any radioprotective effect on colonic tumor tissue. **a** (i) BCN057 treatment did not rescue human malignant colonic organoids from radiation toxicity. Organoids derived from surgical specimens of colon tumor were exposed to irradiation (IR; 8 Gy) and then treated with BCN057. Note the loss of the budding crypt-like structure in irradiated organoids treated with BCN057. Treatment with BCN057 in unirradiated organoids also showed the loss of the budding crypt-like structure, indicating that BCN057 has an inhibitory effect on the growth and proliferation of malignant organoids. (ii) The effect of BCN057 treatment on the growth of irradiated crypt organoids developed from human colon tumor. Budding crypts to total crypt ratio reduced in a dose-dependent manner following irradiation (2–8 Gy). A similar pattern of budding crypts to total crypt ratio was observed in irradiated organoids with BCN057 treatment, indicating that BCN057 could not reduce the radiation toxicity in malignant colonic organoids. **b** BCN057 does not have radioprotective effect on mice abdominal tumors. (i) Representative image of C57BL/6 mice having MC38 colon tumor in the flank at day 20 post-abdominal irradiation (AIR). Note the reduction in tumor size in irradiated or unirradiated tumor treated with BCN057. Mice exposed to AIR without BCN057 treatment are not included in this image as they died within 12 days of AIR. (ii) Tumor growth curve demonstrating the effect of BCN057 treatment on mice abdominal MC38 tumors. Note the significant reduction in tumor growth in BCN057-treated mice following AIR compared with unirradiated untreated controls (*p* < 0.0004). BCN057 treatment in unirradiated mice also reduces the tumor growth compared with unirradiated untreated controls (*p* < 0.0007; *n* = 10/group). The tumor growth curve in the AIR group was discontinued as all mice died from radiation toxicity by day 12 post-radiation. (iii) Kaplan-Meier survival analysis of C57BL/6 mice (*n* = 10/group) with MC38 abdominal tumors receiving AIR, AIR + BCN057, BCN057, or no treatment. Please note that in the AIR control group all the mice died within 12 days post-radiation. Therefore, the tumor growth curve (ii) is not complete in these mice. Mice receiving BCN057 after 15 Gy AIR demonstrated 60% survival beyond day 20 post-irradiation (*p* < 0.0006; log-rank (Mantel-Cox) test). Untreated unirradiated control mice (blue line in the graph) were euthanized on day 20 as their tumor volume reached 2000 mm^3^ (the upper limit of tumor volume approved by the IACUC)

BCN057 was first examined in the National Cancer Institute (NCI) 60 Cancer Cell Line platform [23], which includes the colon cancer cell lines HCT166, HCT15, COLO205, KM12, HT29, SW-620, and HCC-2998. Several of these cell lines are known to be positive for dysregulation of the Wnt/β-catenin signaling pathway. None of these cells showed any proliferative response to BCN057 treatment in our study (Additional file 6: Table S3). We also examined the effect of BCN057 on human colonic tumor-derived organoids exposed to irradiation. Surgical specimens of malignant colonic tissue were obtained from the same individual from whom we collected non-malignant tissue. Treatment with BCN057 (10 μM) at 1 h post-radiation exposure (8 Gy) did not rescue organoids from radiation toxicity. All the budding crypts disappeared within 72–96 h post-irradiation in both BCN057-treated and untreated organoids (Fig. 5a). Moreover, there was no difference in budding crypts/total crypt ratio in BCN057-treated and untreated organoids (Fig. 5a), suggesting that BCN057 does not have a radioprotective effect in malignant tissue-derived organoids.

Subcutaneous tumors were developed by injecting MC38 colon cancer cells into the mice flanks (Fig. 5b). Mice with palpable subcutaneous tumor were exposed to AIR (15 Gy) followed by treatment with or without eight doses of BCN057. AIR alone produced 100% mortality of animals within 12 days (Fig. 5b) of radiation exposure. Therefore, the tumor growth could not be studied in these mice beyond day 12. Compared with AIR alone, mice receiving BCN057 post-AIR showed a significant improvement in survival time (Fig. 5b). In the AIR + BCN057 treated group, 60% of mice survived beyond 20 days post-radiation exposure (Fig. 5b) and showed significant tumor growth retardation compared with untreated and non-irradiated controls (*p* < 0.0004; *n* = 10; Fig. 5b). MC38 colon cancer cells are positive for Wnt-β-catenin signaling [30]. Immunohistochemical analysis of MC38 tumor tissue from untreated non-irradiated mice showed β-catenin-positive nuclei (Additional file 7: Figure S3). However, tumor tissue from BCN057 and AIR + BCN057 did not show the presence of β-catenin-positive nuclei (Additional file 7: Figure S3) indicating that BCN057 failed to activate β-catenin signaling in the MC38 colon tumor.

These results clearly suggest that BCN057 has no protective effect on tumors during radiation therapy and therefore BCN057 may have use in combination therapy to minimize the toxic side-effects of abdominal radiotherapy.

## Discussion

A higher self-renewal rate of ISC makes intestinal epithelium very sensitive to high doses of irradiation. Therefore, it is critical to mitigate radiation-induced gastrointestinal injury to overcome acute radiation syndrome. The present study indicates that treatment with BCN057 starting at 24 h post-abdominal irradiation induces repair and regeneration of intestinal epithelium and improves survival against lethal doses of irradiation. Moreover, BCN057 also rescued mice from RIGS when 40% BM was exposed along with radiation to the intestine, which indicates that BCN057 can partially substitute the radioprotective role of the BM in GI injury.

BCN057 promotes the regenerative response of Lgr5-positive ISC to mitigate RIGS. These data have been replicated in intestinal organoid cultures from Lgr5-EGFP-Cre-ERT2 mice designed to examine the role of Lgr5-positive ISC in stem cell regeneration. This study along with the intestinal organoid culture developed from patient-derived non-malignant colonic epithelium demonstrated that BCN057 induces ISC regeneration. Intestinal epithelial homeostasis and regeneration depends upon Wnt-β-catenin signaling. BCN057 is a small molecular agent which activates Wnt-β-catenin signaling as demonstrated in TCF/LEF luciferase assay as well as in irradiated crypt where it induces the nuclear localization of β catenin. These observations clearly indicate that BCN057 is an agonist of Wnt-β-catenin signaling, and it can rescue the normal epithelial pathology with a resultant survival of mice; this suggests that BCN057 might be an effective mitigator of RIGS.

Intestinal crypts have two types of stem cells. Bmi1-positive ISC are long-lived, label-retaining stem cells present at the +4 position of the crypt base [11]. These Bmi1-positive ISC interconvert with more rapidly proliferating Lgr5-positive stem cells known as crypt base columnar cells (CBCs) [31] that express markers including Lgr5, Olfm4, Lrig1, and Ascl2 [32–34]. These CBCs are also active stem cells and are primarily involved in self-renewal and differentiation. Our previous observation demonstrated that activation of these stem cells post-radiotherapy is critical for repair and regeneration of intestinal epithelium [4]. We have also demonstrated that supplementation of Wnt ligands is critical for activating Wnt-β-catenin signaling and rescuing these stem cells following radiation injury [3]. In the present study, we have demonstrated that BCN057 as a small molecular agent activates Wnt-β-catenin signaling and rescues these ISC from radiation toxicity.

Identification of a suitable animal model to study RIGS and test the candidate agents as mitigators is still a major challenge as the mechanisms underlying this symptom may vary between models. Thus far, multiple animal models have been used to study RIGS, including mice, mini-pigs, canine and non-human primates (NHPs). However, we are not aware of reports describing the testing of radio-mitigators in healthy human tissues to re-affirm the translational relevance of the mechanism from animal studies. In this study, we have used ex-vivo organoids developed from colonic epithelium from human donors and demonstrated that BCN057 induces human colonic stem cell growth and proliferation following radiation. Intestinal organoids retain the crypt villus structure along with all the major cell types of the intestinal epithelium, including ISC, paneth cells, enteroendocrine cells, and enterocytes [19]. Importantly, organoid growth primarily depends on the presence of stem cells [19]. Therefore, this organoid system provides a perfect platform to examine and validate the efficacy of any potential GI radio-mitigators in human tissue and to validate mechanistically the relationship between animal data and their translational value to human tissue.

BCN057 does not have any radioprotective effect on organoids derived from human colon tumors or in mouse subcutaneous tumors. This is consistent with an NCI 60 Cancer Cell Line screen of colon, breast, prostate, ovary, lung, kidney, central nervous system, pancreas, skin and blood, several of which are Wnt-positive cell lines where no enhancement of growth was noted with the application of BCN057. However, some cell lines showed significantly inhibited growth in the presence of the drug. We have observed that BCN057 treatment reduces the proliferation of colon cancer cell lines HCT166, HCT15, COLO205, KM12, HT29, SW-620, and HCC-2998 (Additional file 6: Table S3) where several of these cell lines have Wnt signaling upregulated [35].

Cancer cells in general are more resistant to apoptosis by acquiring mutations in genes, such as p53, or inducing anti-apoptotic genes [36]. Upon genotoxic stress, normal cells with intact p53 undergo apoptotic cell death and can be rescued by inhibition of apoptosis, whereas tumor cells are non-responsive to inhibition of apoptosis and more prone to senescence [37]. Thus, the anti-apoptotic role of BCN057 could protect the ISC by reducing radiation-induced apoptosis but it does not appear to affect the colon cancer cells, which undergo cell death, or senescence, after radiation exposure. Therefore, during radiation therapy, systemic use of BCN057 may be useful in patients undergoing abdominal irradiation for GI malignancies. Clinically, radiation enteritis is a response to the damage to the small and large bowel that occurs with radiation therapy to the pelvic, abdominal, or rectal areas, with about 15–20% of patients requiring an altered therapeutic course [38]. Chronic conditions due to radiation enteritis can present within 1.5 to 6 years after radiotherapy, with some reported up to 30 years later [39], and upwards of 90% of the patients who receive pelvic radiotherapy develop a permanent change in their bowel habit [40]. Up to half of these patients describe their quality of life as being adversely affected by a variety of GI symptoms [41–43] with a significant portion scoring the effect as moderate or severe [44]. Loss of ISC due to acute radiation injury impairs the repair process and promotes the radiation enteritis at later time points [45, 46]. Our data suggest that BCN057 may inhibit radiation enteritis by mitigating the acute effects of radiation with activation of the repair process.

Several growth factors and cytokines, such as KGF, transforming growth factor (TGF)β, tumor necrosis factor (TNF)α, prostaglandin (PG)E2, and interleukin (IL)-11 [47–49], including Wnt agonist R-spondin1, protect the intestine from radiation injury when applied before radiation exposure. However, so far there are no reports on growth factors that can mitigate intestinal injury when applied after radiation exposure. To our knowledge, this is the first demonstration of the salutary effect of BCN057 in the context of radiation injury of the intestine where it mitigates RIGS when applied 24 h after exposure to lethal doses of radiation.

## Conclusions

The present study demonstrates that BCN057 treatment at 24 h post-irradiation exposure rescues Lgr5-positive ISC from radiation-induced loss and promotes epithelial repair and regeneration to mitigate RIGS. However, BCN057 did not have any radioprotective effect in abdominal tumors and therefore could improve the therapeutic efficacy of abdominal radiotherapy.

## Additional files

Additional file 1: Supplement method. Detailed methods of histopathology, immunohistochemistry to determine crypt proliferation rate, β-catenin immunohistochemistry of mouse jejunum, and real-time PCR are described. (DOC 27 kb)

Additional file 2: **Table S1.** β-catenin target gene specific real-time PCR primers (mouse). (DOC 31 kb)

Additional file 3: **Table S2.** Stem cell marker genes and primer sequences (human). (DOC 29 kb)

Additional file 4: **Figure S1.** BCN057 treatment at 72 h post-irradiation could not induce repair of intestinal epithelium. H&E stained representative section of jejunum from C57BL/6 mice treated with BCN057 at 72 h post-AIR. Note the significant damage to intestinal epithelium in both BCN057-treated and untreated mice. (DOC 306 kb)

Additional file 5: **Figure S2.** Confocal microscopic images (×40) of the jejunal section from Lgr5-eGFP-IRES-CreERT2; Rosa26-CAG-tdTomato mice. tdTomato (tdT)-positive cells are shown in red; Lgr5-positive/GFP-positive cells are shown in green. Nuclei are stained with DAPI (blue). (DOC 388 kb)

Additional file 6: **Table S3.** Cancer cell proliferation in the presence of BCN057 10 μM. Table of cells tested at 10 μM BCN057 in neat DMSO on the indicated cell lines representing various cancer types. Values are represented as a percentage of control growth which is the vehicle alone (DMSO). (DOC 26 kb)

Additional file 7: **Figure S3.** Representative microscopic images (×20 magnification) of MC38 colon tumor sections immunostained with anti-β-catenin antibody to determine β-catenin nuclear localization. Please note the absence of β-catenin-positive nuclei in the AIR + BCN057 group. BCN057 treatment in non-irradiated tumors also did not demonstrate β-cateninpositive nucleis. (DOC 461 kb)

## Abbreviations

AIR: Abdominal irradiation
ARS: Acute radiation syndrome
BM: Bone marrow
CBC: Crypt base columnar cell
EV: Extracellular vesicle
GI: Gastrointestinal tract
H&E: Hematoxylin and eosin
ISC: Intestinal stem cells
LiCl: Lithium chloride
NHP: Non-human primate
PBI: Partial body irradiation
PBS: Phosphate-buffered saline
PCR: Polymerase chain reaction
PK: Pharmacokinetics
qPCR: Quantitative polymerase chain reaction
RIGS: Radiation-induced gastrointestinal syndrome
RSPO1: R-spondin 1
s.c.: Subcutaneous
TUNEL: TdT-mediated digoxigenin-labeled dUTP nick-end labeling
