# Supplementary material for: BCN057 induces intestinal stem cell repair and mitigates radiation-induced intestinal injury
Source: Stem Cell Res Ther. 2018 Feb 2;9:26. doi: 10.1186/s13287-017-0763-3 (PMC5797353; doi:10.1186/s13287-017-0763-3)
Supplement: Supplementary file 1 — Supplement method. Detailed methods of histopathology, immunohistochemistry to determine crypt proliferation rate, β-catenin immunohistochemistry of mouse jejunum, and real-time PCR are described. (DOC 27 kb) [file 13287_2017_763_MOESM1_ESM.doc]

**Supplement method:**

**Histology**

The intestine of each animal was dissected, washed in PBS to remove intestinal contents and the jejunum was fixed in 10% neutral-buffered formalin before paraffin embedding. Tissue was routinely processed and cut into 5 μm sections for haematoxylin and eosin and immunohistochemical staining. All haemotoxylin and eosin (HE) (Fisher Scientific, Pittsburgh, PA) staining was performed at the Pathology Core Facility in the KUMC Cancer Center.

**Immunohistochemistry to determine crypt proliferation rate**

Digital photographs of crypts were taken at high (× 20–60) magnification (Zeiss AxioHOME microscope) and crypt epithelial cells in intestinal sections were examined using ImageJ software and classified as Ki67 positive if they grossly demonstrated brown-stained nuclei from DAB staining or as Ki67 negative if they were blue stained nuclei. The proliferation rate was calculated as the percentage of Ki67-positive cells over the total number of cells in each crypt. A total of 60 crypts were examined per animal.

**β-catenin immunohistochemistry of mouse jejunum**

β-catenin immunohistochemistry was performed in paraffin-embedded sections of mouse jejunum [16]. Before immunostaining, antigen retrieval was performed by heating slides in pH 6.0 citrate buffer at 100 °C for 20 min in a microwave oven at 500 W using antigen retrieval solution (10 mM Tris and 1 mM EDTA, pH 9.0). Non-specific antibody binding was blocked for 20 min by incubation with 0.05% w/v BSA in PBS. Tissue was stained using the anti-β-catenin antibody (1:100 dilution; BD Transduction Laboratories, Franklin Lakes, NJ; #610154) at room temperature for 2hr followed by staining with horseradish peroxidase-conjugated Anti-Mouse Antibody (Dako, Denmark) at room temperature for 1 h. Peroxidase activity was detected by adding DAB substrate. Nucleus was counter-stained with haematoxylin (blue). β-Catenin-positive nucleus (stained dark brown) was calculated from 15 crypts per field, 5 fields per mice.

**Real-time PCR**

Total RNA was extracted using TRIzol kit (Invitrogen, CA). RNA was reverse transcribed in a final volume of 20 μL using 0.5 μg of oligo dT and 200 U Superscript III RT (Invitrogen) for 30 min at 50°C, followed by 2 min at 94°C to inactivate the reverse transcriptase. Real-time PCR amplification was carried out in a total volume of 25 μL containing 0.5 μM of each primer, 4 mM MgCl2, 12.5 μL of LightCycler™ FastStart DNA Master SYBR green I (Roche Molecular Systems, Alameda, CA) and 10 μL of 1:20 diluted cDNA. PCR reactions were prepared in duplicate and heated to 95°C for 10 min followed by 40 cycles of denaturation at 95°C for 15 sec, annealing at 60°C for 1 min, and extension at 72°C for 20 sec in ABI Applied Biosystems™ QuantStudio™ Real time PCR machine. Standard curves (cycle threshold values versus template concentration) were prepared for each target gene and for the endogenous reference (GAPDH) in each sample. Quantification of the unknown samples was performed using the nanodrop (Thermofisher scientific).
