# Supplementary material for: BCN057 induces intestinal stem cell repair and mitigates radiation-induced intestinal injury
Source: Stem Cell Res Ther. 2018 Feb 2;9:26. doi: 10.1186/s13287-017-0763-3 (PMC5797353; doi:10.1186/s13287-017-0763-3)
Supplement: Supplementary file 2 — Table S1. β-catenin target gene specific real-time PCR primers (mouse). (DOC 31 kb) [file 13287_2017_763_MOESM2_ESM.doc]

**Table S1**: β-catenin target gene specific real-time PCR Primers.

| Gene name | Forward Primer | Reverse Primer |
| --- | --- | --- |
| Ephb2 | 5′ACGCCACGGCCATAAAAAGCCC-3′ | 5′-TTGCCACTGTAGCGCCCATAGC-3′ |
| Ascl | 5′-GGAGCAGGAGCTGCTTGACT -3′, | - 5′-AGGTAGGTCCACCAGGAGTCA -3′ |
| Lef | 5′-AAATGGGTCCCTTTCTCCAC -3′, | 5′-TCGTCGCTGTAGGTGAGAAG 3” |
| Sox9 | 5′-CTCGCATACCTCCCTTCC-3′ | 5′-TTCCAGCAGTCACTAGGC-3′ |
| Axin | 5′-GGACTGGGGAGCCTAAAGGT-3′ | 5′-AAGGAGGGACTCCATCTACGC-3′ |
| TCF4 | 5′-CTGCCTTAGGGACGGACAAAG-3′ | 5′-TGCCAAAGAAGTTGGTCCATTTT-3′ |
| Olf | 5′-GCCACTTTCCAATTTCAC-3′ | 5′-GAGCCTCTTCTCATACAC-3′ |

­­
