# Supplementary material for: BCN057 induces intestinal stem cell repair and mitigates radiation-induced intestinal injury
Source: Stem Cell Res Ther. 2018 Feb 2;9:26. doi: 10.1186/s13287-017-0763-3 (PMC5797353; doi:10.1186/s13287-017-0763-3)
Supplement: Supplementary file 3 — Table S2. Stem cell marker genes and primer sequences (human). (DOC 29 kb) [file 13287_2017_763_MOESM3_ESM.doc]

**Table S2**: Stem cell marker genes and primer sequences (Human)

| Gene name | Forward Primer | Reverse Primer |
| --- | --- | --- |
| Lgr5 | 5′-TCAGTCAGCTGCTCCCGAAT-3′ | 5′-CGTTTCCCGCAAGACGTAAC-3 |
| K19 | 5’ CTTCCGAACCAAGTTTGAGAC 3’ | 5’ A5′-GCGTACTGATTTCCTCCTC 3’ |
| Hes-1 | 5'-ATGGAGAAAAATTCCTCGTCCC-3' | 5'-TTCAGAGCATCCAAAATCAGTGT-3' |
| CD44 | 5′-TCCAACACCTCCCAGTATGACA-3′ | ′-GGCAGGTCTGTGACTGATGTACA-3′; |
| GAPDH | 5’TCAGTTGTAGGCAAGCTGCGACGT | 5’AAGCCAGAGGCTGGTACCTAGAAC 3’ |
