# Supplementary figures and images for: BCN057 induces intestinal stem cell repair and mitigates radiation-induced intestinal injury
Source: Stem Cell Res Ther. 2018 Feb 2;9:26. doi: 10.1186/s13287-017-0763-3 (PMC5797353; doi:10.1186/s13287-017-0763-3)

**Figure S1**

**
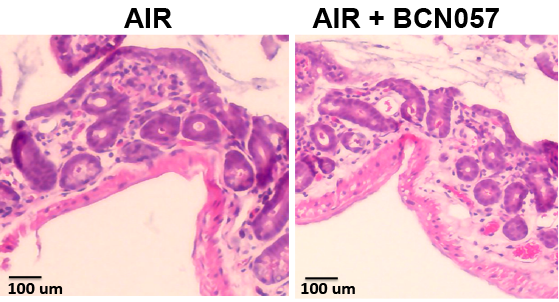
**

Supplement: Supplementary file 4 — Figure S1. BCN057 treatment at 72 h post-irradiation could not induce repair of intestinal epithelium. H&E stained representative section of jejunum from C57BL/6 mice treated with BCN057 at 72 h post-AIR. Note the significant damage to intestinal epithelium in both BCN057-treated and untreated mice. (DOC 306 kb) [file 13287_2017_763_MOESM4_ESM.doc]

**Figure S2:**

**
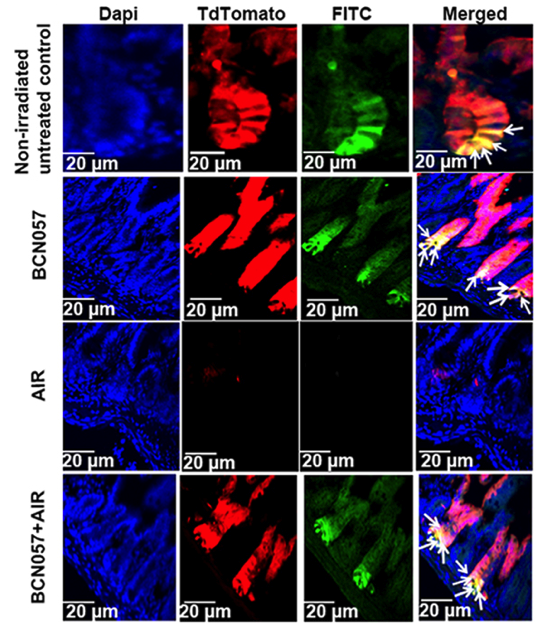
­­**

Supplement: Supplementary file 5 — Figure S2. Confocal microscopic images (×40) of the jejunal section from Lgr5-eGFP-IRES-CreERT2; Rosa26-CAG-tdTomato mice. tdTomato (tdT)-positive cells are shown in red; Lgr5-positive/GFP-positive cells are shown in green. Nuclei are stained with DAPI (blue). (DOC 388 kb) [file 13287_2017_763_MOESM5_ESM.doc]

# Figure S3:

**
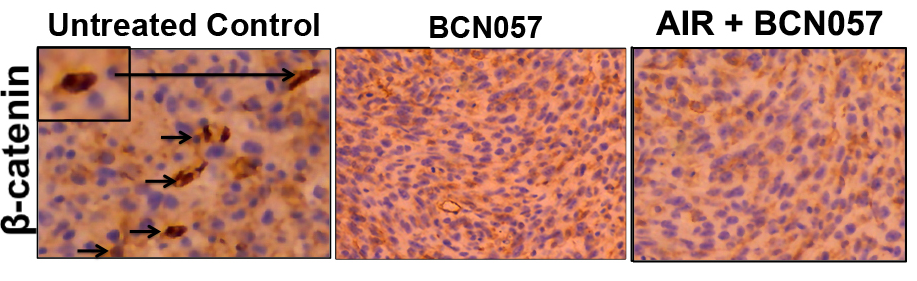
**

Supplement: Supplementary file 7 — Figure S3. Representative microscopic images (×20 magnification) of MC38 colon tumor sections immunostained with anti-β-catenin antibody to determine β-catenin nuclear localization. Please note the absence of β-catenin-positive nuclei in the AIR + BCN057 group. BCN057 treatment in non-irradiated tumors also did not demonstrate β-cateninpositive nucleis. (DOC 461 kb) [file 13287_2017_763_MOESM7_ESM.doc]
