# Supplementary material for: BCN057 induces intestinal stem cell repair and mitigates radiation-induced intestinal injury
Source: Stem Cell Res Ther. 2018 Feb 2;9:26. doi: 10.1186/s13287-017-0763-3 (PMC5797353; doi:10.1186/s13287-017-0763-3)
Supplement: Supplementary file 6 — Table S3. Cancer cell proliferation in the presence of BCN057 10 μM. Table of cells tested at 10 μM BCN057 in neat DMSO on the indicated cell lines representing various cancer types. Values are represented as a percentage of control growth which is the vehicle alone (DMSO). (DOC 26 kb) [file 13287_2017_763_MOESM6_ESM.doc]

**Table S3**:

**Cancer Cell Proliferation in the presence of BCN057 10μM**
